# Persistent and aggressive interactions with the police: potential mental health implications

**DOI:** 10.1017/S2045796019000015

**Published:** 2019-02-05

**Authors:** J.L. Hirschtick, S.M. Homan, G. Rauscher, L.H. Rubin, T.P. Johnson, C.E. Peterson, V.W. Persky

**Affiliations:** 1 Sinai Health System, Sinai Urban Health Institute, 1500 S. California Ave K443, Chicago, IL 60608, USA; 2 University of Illinois at Chicago, School of Public Health, 1603 W. Taylor Street, Chicago, IL 60612, USA; 3 Department of Psychiatry, University of Illinois at Chicago, 912 S. Wood Street, Chicago, IL 60612, USA; 4 University of Illinois at Chicago, Survey Research Laboratory, 1200 W. Harrison Street, Chicago, IL 60607, USA

**Keywords:** Depression, discrimination, epidemiology, population survey, post-traumatic stress disorder

## Abstract

**Aims:**

Little is known about the potential health impact of police encounters despite a ubiquitous police presence in many disadvantaged urban environments. In this paper, we assess whether persistent or aggressive interactions with the police are associated with poor mental health outcomes in a sample of primarily low-income communities of colour in Chicago.

**Methods:**

Between March 2015 and September 2016, we surveyed 1543 adults in ten diverse Chicago communities using a multistage probability design. The survey had over 350 questions on health and social factors, including police exposure and mental health status. We use sex-stratified logistic regression to examine associations between persistent police exposure (defined as a high number of lifetime police stops) or aggressive police exposure (defined as threat or use of police force during the respondent's most recent police stop) and the presence of post-traumatic stress disorder (PTSD) or depressive symptoms.

**Results:**

Men reporting a high number of lifetime police stops have three times greater odds of current PTSD symptoms compared with men who did not report high lifetime police stops (OR 3.1, 95% CI 1.3–7.6), after adjusting for respondent age, race/ethnicity, education, history of homelessness, prior diagnosis of PTSD and neighbourhood violent crime rate. Women reporting a high number of lifetime police stops have two times greater odds of current PTSD symptoms, although the results are not statistically significant after adjustment (OR 2.0, 95% CI 0.9–4.2). Neither persistent nor aggressive police exposure is significantly associated with current depressive symptoms in our sample.

**Conclusions:**

Our findings support existing preliminary evidence of an association between high lifetime police stops and PTSD symptoms. If future research can confirm as causal, these results have considerable public health implications given the frequent interaction between police and residents in disadvantaged communities in large urban areas.

## Introduction

Aggressive policing tactics, such as stop-and-frisk and excessive use of force, routinely occur in many urban environments, particularly disadvantaged communities of colour (Ayres and Borowsky, 2008; American Civil Liberties Union of Illinois, 2015; Department of Justice, 2017). Such tactics can trigger mistrust and emotional distress among marginalised populations that disproportionately experience them, including racial/ethnic minority groups and immigrants (Fagan and Davies, 2000; Brunson, 2007; Ayres and Borowsky, 2008; Brunson and Weitzer, 2009; American Civil Liberties Union of Illinois, 2015). Yet little is known about the potential health impact of aggressive policing. Persistent or aggressive police exposure may have detrimental effects on an individual's mental health status.

There are several plausible theoretical pathways between policing exposure and mental health status. Persistent exposure to the police may be perceived as discrimination, which is associated with a number of negative mental health outcomes (Williams and Williams-Morris, 2000; Pascoe and Smart Richman, 2009; Sawyer *et al*., 2012; Freeman Anderson, 2013) and can operate at several levels (Williams and Williams-Morris, 2000). At the institutional level, government policies result in heightened police presence in disadvantaged communities. Several studies have found that racial/ethnic minorities are stopped by the police at disproportionately high rates even after adjusting for neighbourhood crime, suggesting a discriminatory mechanism (Fagan and Davies, 2000; Ayres and Borowsky, 2008). At the individual level, negative stereotypes may be internalised, leading to feelings of lower self-esteem and self-worth (Williams and Williams-Morris, 2000). Furthermore, repeated exposure to discrimination may increase stress levels, activating a perpetual stress response (Williams and Williams-Morris, 2000; Sawyer *et al*., 2012).

Police violence may also impact mental health via the trauma pathway. Exposure to aggressive policing tactics, such as use of force, may traumatise the victim, thereby increasing the risk of a number of mental disorders (Health and Human Services, 1999). Additionally, chronic stress has been linked to mental health outcomes including depression (Hammen, 2005) and anxiety disorders (Faravelli *et al*., 2012) via the hypothalamic pituitary adrenal axis. If individuals perceive recurrent police exposure as stressful, dysregulation of the body's natural response to stress may result in prolonged secretion of cortisol leading to allostatic load (McEwen, 1998).

Nearly two decades ago, Williams and Williams-Morris recognised that a ‘high level of police surveillance may be an important but neglected source of stress in the lives of many African Americans’ (Williams and Williams-Morris, 2000). Yet the field of public health has been slow to respond to requests for analyses of the health consequences of aggressive policing (Williams and Williams-Morris, 2000; Cooper *et al*., 2004; Krieger *et al*., 2015). In 2017, the American Public Health Association issued a policy statement, *Addressing Law Enforcement Violence as Public Health Issue* (Rubin *et al*., 2017). This statement, along with an emerging body of empirical evidence, links persistent and aggressive policing tactics, such as frequent police stops or police-perpetrated violence, to symptoms of depression (DeVylder *et al*., 2016; English *et al*., 2017), anxiety and post-traumatic stress disorder (PTSD) (Geller *et al*., 2014), psychological distress (DeVylder *et al*., 2016; Sewell *et al*., 2016) and suicide attempts (DeVylder *et al*., 2017). One recent study found that police killings of unarmed black Americans were associated with the number of poor mental health days for black survey respondents in the months immediately following these events, thereby providing preliminary evidence that negative consequences of police violence may extend beyond those directly involved (Bor *et al*., 2018).

Despite this recent attention, a focused research effort is needed to increase our understanding of this important public health issue. We are only aware of one population-based study on this topic to date, which was limited to young men in New York City (Geller *et al*., 2014). Chicago, a hyper-segregated city with a history of police brutality (Massey and Denton, 1989; Chicago Tribune, 2018), provides a unique environment in which to examine this issue.

In this paper, we seek to build on preliminary evidence to better understand the impact of police encounters on the mental health of individuals living in economically and socially disadvantaged communities. Our hypothesis is that both persistent and aggressive police encounters may negatively impact current mental health status, through either the frequency or violent nature of these encounters. Using a population-based survey, we examine associations between persistent or aggressive police exposure, measured as number of lifetime police stops or threat or use of force during the respondent's most recent police stop, respectively, with current PTSD and depressive symptoms among men and women in several diverse Chicago communities.

## Methods

### Data collection

The Sinai Community Health Survey 2.0 (Sinai Survey) was a cross-sectional, multistage probability health survey of selected Chicago community areas led by Sinai Urban Health Institute (SUHI), a member of Sinai Health System in Chicago. Community areas are a geographic designation unique to Chicago composed of contiguous census tracts. Under SUHI's direction, the University of Illinois at Chicago's Survey Research Laboratory (SRL) interviewed residents in ten Chicago community areas (Chicago Lawn, Gage Park, Hermosa, Humboldt Park, Lower West Side, North Lawndale, Norwood Park, South Lawndale, west-West Town and West Englewood) from March 2015 through September 2016. As part of the original sampling structure, we selected community areas based on geographic location and racial/ethnic composition to include Puerto Rican, Mexican, Non-Hispanic (NH) black and NH white residents. Except for Lower West Side (*n* = 33) and west-West Town (western portion only), each community's sample (ranging from *n* = 88–245) is representative of its respective community area population (see Appendix Table A1).

Within each community area, SRL randomly selected census block clusters, households and household residents, under-sampling hard-to-reach households (e.g. households with a doorman or locked gates). Interviewers made at least ten in-person attempts to contact selected individuals. One or two adult respondents (aged 18 and over) were selected to complete the adult questionnaire based on a random selection algorithm. The survey instrument included over 350 questions on a number of health topics and took 84 min to complete on average. SRL interviewed 1543 respondents for the adult survey in both English and Spanish using Computer Assisted Personal Interviewing (CAPI).

The response rate, calculated by dividing the number of completed interviews by the number of interviews, refusals, non-contacts of known eligible respondents and a proportion of households with unknown eligibility (per American Association for Public Opinion Research (AAPOR) response rate formula 3), was 28.4% (American Association for Public Opinion Research, 2016). The cooperation rate, defined as the number of completed interviews divided by the number of interviews and refusals (per AAPOR cooperation rate formula 4), was 53.9%. Sinai Survey data collection and analysis was approved by the Institutional Review Boards at University of Illinois at Chicago (Protocol #2014-0524) and Mount Sinai Hospital (IRB-MSH #14-17).

### Measures

We conceptualised police exposure in terms of persistence, measured by number of lifetime police stops, and aggressiveness, measured by perceived threat or use of force during the respondent's most recent police stop. Respondents answered questions on police experiences based on the Police Public Contact Survey, a supplement to the National Crime Victimization survey conducted by the Bureau of Justice Statistics in the Department of Justice (Department of Justice Bureau of Justice Statistics, 2017). We used the following question to measure lifetime police stops: ‘How many times have you been stopped by the police during your lifetime?’ We did not further define police stop in the questionnaire, so responses may have included any police encounter viewed as a stop by the respondent (e.g. street stop, traffic stop, etc.). Responses were top-coded at 21 stops or more at the time of data collection.

*A priori*, we conceptualised a potential threshold effect rather than a linear relationship between lifetime police stops and mental disorder symptoms. Small cell sizes precluded us from categorising police stops in a conceptually meaningful way. Instead, we dichotomised lifetime police stops as high *v.* low using sex-specific 75^th^ percentiles (>3 for women and >15 for men).

We classified respondents as exposed to police use of force if they answered yes to the following question: ‘Please tell me ONLY about the MOST RECENT time the police stopped you. During this stop, did the police use or threaten to use force against you for any reason?’ Respondents who had never been stopped by the police, and therefore never experienced threat or use of police force, were considered unexposed.

The survey included screenings for current PTSD and depressive symptoms. An abbreviated version of the Post-Traumatic Stress Disorder Checklist for Civilians (PCL-C) measured current PTSD symptoms (Weathers *et al*., 1993; Blanchard *et al*., 1996). The six-item PCL-C is based on PTSD symptom criteria in the Diagnostic Statistical Manual of Mental Disorders (DSM) version IV (American Psychiatric Association, 2000), including hyper-arousal, re-experiencing and avoidance symptom clusters. Respondents reported how much a symptom affected them in the past 30 days, with five ordinal responses ranging from not at all (1 point) to extremely (5 points). Since the distribution of PCL-C scores was highly positively skewed, we used the previously established cut-point of 14 (out of 30 possible points) to represent the presence of current PTSD symptoms (Lang and Stein, 2005; Lang *et al*., 2012).

The nine-item Patient Health Questionnaire (PHQ-9) (Spitzer *et al*., 1999), a validated and widely-used screening tool (Martin *et al*., 2006; Merz *et al*., 2011; Kocalevent *et al*., 2013), captured depressive symptoms in the past 2 weeks based on the nine criteria for depression diagnosis from the DSM version IV. The PHQ-9 asks respondents to indicate the frequency with which each symptom has bothered them in the past 2 weeks, ranging from not at all (0 points) to nearly every day (3 points). Since the PHQ-9 score distribution in our sample was also highly positively skewed, we used the previously established cut-point of ten (out of 27 points possible) to represent the presence of current depressive symptoms (Kroenke *et al*., 2001; Centers for Disease Control and Prevention, 2011; Merz *et al*., 2011).

### Statistical analysis

We used survey commands in Stata version 14 (StataCorp, 2015) to account for the complex sampling design of Sinai Survey, including clustering and stratification. Weights addressed the probability of respondent selection and aligned each community area's sample with the racial/ethnic, sex and age distribution of its respective target population.

**Fig. 1.**
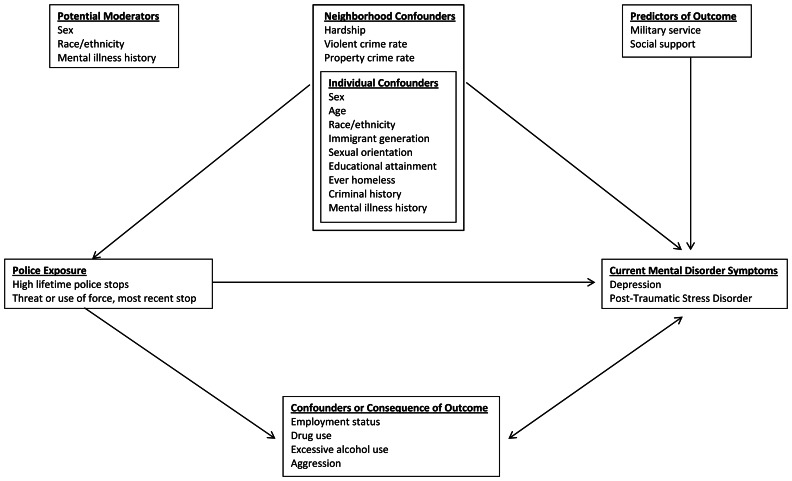
Conceptual framework: Cross-sectional associations between police encounters and mental health status.

Our conceptual framework guided the modelling strategy (Fig. 1). We chose a group of consistent confounders for all adjusted models based on *a priori* knowledge (age, race/ethnicity, educational attainment, previous diagnosis of depression or PTSD and neighbourhood violent crime rate) or empirical evidence of a strong association with both the exposures and outcomes (ever homeless). All confounders were pulled from Sinai Survey 2.0 apart from neighbourhood violence crime rate. We calculated violent crime rate per 1000 persons at the census tract level by dividing crime counts for the period of March 2016–March 2017 from the Chicago Police Department's CLEARMAP crime summary reporting tool (Chicago Police Department, 2017) by the corresponding census tract population. We also fit a series of over-adjusted models that included confounders from the adjusted models and a set of variables that may be confounders, on the aetiologic pathway, or consequences of current mental health status (employment status, drug use, excessive alcohol use and aggressiveness).

We conducted sex-stratified logistic regression to account for different levels of police exposure and distinct mental disorder risk profiles for men and women. We additionally tested potential interactions between the police exposure variables and both race/ethnicity and mental illness history. However, due to small cell sizes, we limit the presentation of our findings to estimates of the main effects with the understanding that these relationships may be more nuanced and require a larger sample size to understand possible interactions by race/ethnicity and mental illness history. We also conducted a sensitivity analysis with a continuous version of lifetime police stops.

## Results

### Descriptive statistics

Weighted descriptive statistics for the study population are summarised in Table 1. Residents in surveyed communities face a number of hardships. Eighteen per cent were out of work or unable to work at the time of the survey. One in ten respondents had ever been homeless. Twenty-eight per cent reported drug use in the previous year. Nineteen per cent of women and 24% of men had a high number of lifetime police stops (>3 stops for women or >15 stops for men). Twelve per cent of men and 4% of women reported threat or use of force during their most recent police stop, compared with 4 and 2% (respectively) nationally (Davis *et al*., 2018). Twelve per cent of the sample had experienced depressive symptoms in the past 2 weeks, compared with 7% nationally (Centers for Disease Control and Prevention, 2011). Sixteen per cent had experienced PTSD symptoms in the past 30 days, compared with a national annual prevalence of 4% (Kessler *et al*., 2005).

**Table 1.** Summary statistics of Sinai Community Health Survey sample overall and by sex, Chicago, March 2015–September 2016 (*n* = 1543)

	Men, N	Men, median or %	Women, N	Women, median or %	Total, N	Total, median or %
Age		38		42		40
Race/ethnicity						
Puerto Rican	72	6	79	6	151	6
Mexican	225	46	296	40	521	43
Hispanic other	32	4	51	6	83	5
Non-Hispanic Black	214	26	322	33	536	29
Non-Hispanic White	114	17	105	15	219	16
Non-Hispanic other	15	2	14	1	29	1
Immigrant generation						
First	169	33	237	32	406	32
Second	108	23	123	19	231	21
Third or higher	388	45	498	49	886	47
Sexual orientation						
Gay, bisexual or other (non-straight)	23	3	67	6	90	4
Educational attainment						
Less than high school degree	178	27	229	26	407	27
High school degree or GED	375	60	487	57	862	59
College degree or more	119	13	153	17	272	15
Employment status						
Employed for wages	355	55	388	50	743	52
Self-employed	74	11	58	5	132	8
Out of work or unable to work	138	17	192	19	330	18
Homemaker, student or retired	102	17	224	26	326	22
Drug/alcohol use						
Drug use, past year	235	36	176	20	411	28
Excessive alcohol use, past 30 days	175	25	130	14	305	20
Aggression						
In physical fight, past year	80	11	55	6	135	9
Homelessness						
Ever been homeless	91	11	101	10	192	10
Criminal history						
Convicted of criminal charges since age 18	165	24	43	5	208	14
Diagnosed mental illness						
Ever diagnosed with depression	80	10	190	20	270	15
Ever diagnosed with post-traumatic stress disorder	22	2	59	9	81	5
Persistent police encounters						
High number of lifetime police stops	179	24	185	19	364	21
Aggressive police encounter						
Police used or threatened force during most recent stop	90	12	30	4	120	8
Current mental disorder symptoms						
Depressive symptoms, past 2 weeks	54	8	124	15	178	12
Post-traumatic stress disorder symptoms, past 30 days	100	15	169	20	269	16

Percentages do not always sum to 100 due to rounding.

Unweighted sample sizes and weighted estimates are presented.

### Police encounters and PTSD symptoms

Logistic regression results for associations between police encounters and current PTSD symptoms by sex are presented in Table 2. Men with a high number of lifetime police stops (>15) have significantly increased odds of current PTSD symptoms compared with men without a high number of lifetime police stops, an association that remains strong and significant in the adjusted model (OR 3.1, *p* = 0.013) and over-adjusted model. Among women, there is a significant association between a high number of lifetime police stops (>3) and current PTSD symptoms in the unadjusted model (OR 1.9, *p* = 0.049). This association maintains its magnitude but loses statistical significance after adjusting for potential confounders (OR 2.0, *p* = 0.077).

**Table 2.** Adjusted associations for persistent and aggressive police encounters and current post-traumatic stress disorder symptoms, Sinai Community Health Survey, Chicago, March 2015–September 2016^a^

	Men, unadjusted OR (95% CI)	Men, adjusted^b^ OR (95% CI)	Men, over-adjusted^c^ OR (95% CI)	Women, unadjusted OR (95% CI)	Women, adjusted^b^ OR (95% CI)	Women, over-adjusted^c^ OR (95% CI)
*Persistent police encounters*						
High lifetime stops	3.4 (1.6–7.3)*	3.1 (1.3–7.6)*	2.8 (1.2–6.8)*	1.9 (1.0–3.7)*	2.0 (0.9–4.2)	1.7 (0.7–3.8)
*Aggressive police encounter*						
Threat or use of force, most recent stop	2.5 (1.0–6.4)	1.3 (0.6–3.1)	0.9 (0.3–2.2)	3.9 (1.2–12.7)*	2.0 (0.5–7.4)	1.3 (0.3–5.8)

*Statistically significant at *p* ⩽ 0.05.

a Sample size ranged from 653 to 670 for men and 825 to 866 for women depending on covariates.

b Adjusted for respondent age, race/ethnicity, education, history of homelessness, prior diagnosis of PTSD and neighbourhood violent crime rate.

c Additionally adjusted for respondent employment status, drug use, excessive alcohol use and aggressiveness.

In the unadjusted model, men reporting threat or use of force during their most recent police stop have greater odds of current PTSD symptoms than men without perceived threat or use of force during their most recent police stop, although only marginally significant (OR 2.5, *p* = 0.052). This odds ratio is reduced and not significant after adjusting for potential confounders (OR 1.3, *p* = 0.546). Age, which is inversely associated with reported threat or use of force during the most recent police stop and PTSD symptoms for men, and a history of homelessness appear to be important confounders of this relationship. Similarly, the association between perceived threat or use of force during the most recent police stop and current PTSD symptoms among women in the unadjusted model (OR 3.9, *p* = 0.027) is reduced and no longer significant in the adjusted model (OR 2.0, *p* = 0.300) and further reduced in the over-adjusted model. For women, a history of homelessness, race/ethnicity and a prior diagnosis of PTSD all positively confound this association.

### Police encounters and depressive symptoms

Table 3 summarises logistic regression results for associations between police encounters and current depressive symptoms by sex. In the unadjusted model, men with a high number of lifetime police stops have 2.6 times greater odds of current depressive symptoms than men without a high number of lifetime police stops (*p* = 0.011). However, this odds ratio is reduced and no longer statistically significant after adjusting for potential confounders. This is largely driven by joint confounding by a history of homelessness and prior diagnosis of depression. Among women, the relationship between high number of lifetime police stops and current depressive symptoms is not statistically significant in unadjusted or adjusted models (adjusted OR 1.5, *p* = 0.417). The relationship between high lifetime stops and current depressive symptoms in the over-adjusted models is comparable to adjusted model estimates.

**Table 3.** Adjusted associations for persistent and aggressive police encounters and current depressive symptoms, Sinai Community Health Survey, Chicago, March 2015–September 2016^a^

	Men, unadjusted OR (95% CI)	Men, adjusted^b^ OR (95% CI)	Men, over-adjusted^c^ OR (95% CI)	Women, unadjusted OR (95% CI)	Women, adjusted^b^ OR (95% CI)	Women, over-adjusted^c^ OR (95% CI)
*Persistent police encounters*						
High lifetime stops	2.6 (1.2–5.4)*	1.5 (0.7–3.6)	1.3 (0.5–3.1)	1.5 (0.6–3.6)	1.5 (0.5–4.5)	1.5 (0.5–4.5)
*Aggressive police encounter*						
Threat or use of force, most recent stop	1.6 (0.6–3.9)	1.0 (0.3–3.2)	0.5 (0.1–1.8)	3.9 (1.0–14.8)*	2.9 (0.5–17.0)	1.8 (0.4–8.0)

*Statistically significant at *p* ⩽ 0.05.

a Sample size ranged from 632 to 657 for men and 829 to 861 for women depending on covariates.

b Adjusted for respondent age, race/ethnicity, education, history of homelessness, prior diagnosis of depression and neighbourhood violent crime rate.

c Additionally adjusted for respondent employment status, drug use, excessive alcohol use and aggressiveness.

Perceived threat or use of force during the respondent's most recent police stop is not associated with current depressive symptoms among men. However, women reporting threat or use of force during their most recent police stop have nearly four times greater odds of current depressive symptoms than women reporting no threat or use of force during their most recent police stop (OR 3.9, *p* = 0.045). The magnitude of this association decreases and is not statistically significant after adjusting for potential confounders (OR 2.9, *p* = 0.227). A history of homelessness, race/ethnicity, violent crime rate and prior diagnosis of depression appeared to be jointly responsible for reducing the magnitude and significance of this estimate. Over-adjusted models of perceived threat or use of force and current depressive symptoms do not vary substantially from adjusted models, although exposure point estimates are lower in magnitude. We were unable to accurately assess interactions between either police exposure and race/ethnicity or mental illness history as small cell sizes led to unstable estimates.

### Sensitivity analysis

Apart from differences in point estimates from the dichotomised police stop variable, results did not differ in interpretation or significance for associations between continuous lifetime police stops and depression for men or women. For men, the significant positive association between continuous lifetime stops and PTSD symptoms in the unadjusted model becomes marginally significant after adjustment (*p* = 0.060) and no longer significant after potential over-adjustment (*p* = 0.120). For women, the positive association between lifetime police stops and PTSD symptoms is only marginally significant in the unadjusted model when using the continuous exposure variable (*p* = 0.083).

## Discussion

Our findings suggest a potential association between persistent police exposure and current mental health status. High lifetime police stops are strongly and significantly associated with current PTSD symptoms among men, and marginally significantly associated among women. Yet perceived threat or use of force during the most recent police stop is not significantly associated with current PTSD symptoms for men or women after adjustment. It is somewhat unexpected that high lifetime police stops, but not perceived use of force during the most recent stop, is associated with current PTSD symptoms.

This finding, however, is consistent with another population-based study on this subject. Geller *et al*. found a significant association between lifetime police stops and PTSD symptoms among young men in New York City, although their PTSD scale was specific to the respondent's most memorable police stop (Geller *et al*., 2014). One plausible explanation is that exposure to repeated police stops with often unpleasant (e.g. embarrassment, fear) to intolerable (e.g. physical harm, arrests) consequences could result in PTSD symptoms (hyper-arousal, avoidance and re-experiencing). Furthermore, persistent exposure to police authority, especially if perceived as discriminatory, may activate a perpetual stress response (Williams and Williams-Morris, 2000; Sawyer *et al*., 2012).

Alternatively, persistent police stops may be serving as a proxy for lifetime exposure to aggressive policing tactics. In this study, perceived threat or use of force was measured in relation to the *most recent* police stop only. Therefore, we cannot interpret these results as lifetime exposure to aggressive policing. Individuals who have been repeatedly stopped by the police may be at greater risk for exposure to aggressive policing tactics over the course of their lifetime. In this instance, the trauma pathway may be operating, whereby exposure to police aggression or violence may traumatise the victim, leading to symptoms consistent with PTSD.

Reverse causality may also be at play, whereby individuals with existing PTSD may be more likely to be stopped by the police. This is a concern because individuals with mental disorders may be at increased risk of police contact (Hartford *et al*., 2005; Crocker *et al*., 2009). We controlled for previously diagnosed mental illness in our adjusted models in an effort to address this issue, although temporality between previous diagnosis and police exposure also cannot be established in our data. Additionally, most of the surveyed communities are underserved, particularly in the area of mental health care, and may have a non-negligible amount of individuals with undiagnosed mental disorders.

There is not strong evidence of an association between police exposure and depressive symptoms in our sample. High lifetime police stops are associated with current depressive symptoms among men in the unadjusted model, but the odds ratio is diminished and no longer significant after adjustment. Similarly, among women, there is a strong but partially confounded relationship between perceived threat or use of force during the most recent police stop and current depressive symptoms.

### Strengths and limitations

This study advances our knowledge of potential associations between persistent police exposure and current mental health status using a representative population-based survey of several Chicago communities. The Sinai Survey is a unique, comprehensive survey of hard-to-reach populations in Chicago. In addition to collecting information on both police exposures and mental health, Sinai Survey included a large number of related social and demographic factors, allowing for the measurement and control of a number of potentially confounding variables. Given the importance and nature of conducting research in disadvantaged communities, we were pleased to successfully complete interviews over 50% of the time when a household resident was reached.

Precision of our findings is limited by lack of detailed information on the timing of the respondent's most recent police stop, no measure of exposure to aggressive policing tactics beyond the most recent stop, and the inability to distinguish between threat and use of force. Although we asked whether the respondent had been stopped in the past year, we did not restrict the analysis to respondents who reported threat or use of force in the past year as it diminished the proportion exposed considerably. We attempted to alleviate potential recall issues related to reporting lifetime police stops by categorising number of police stops as either high or low using sex-specific percentile cut-points. Additionally, we did not collect information on when depressive or PTSD symptoms first occurred. As a cross-sectional study, we cannot clearly establish temporality.

Additional population-based studies in urban environments are needed to confirm our findings and further knowledge on associations between police encounters and mental health status. Future studies should differentiate between threat and use of force and include a large enough sample size to examine associations by race/ethnic group across different neighbourhood environments. Longitudinal studies are needed to establish temporality and address reverse causality issues, as well as collect detailed information on the timing, nature and type of police encounters experienced. Finally, in addition to studying the impact of police exposure at the individual level, future research should attempt to quantify and assess the impact of police discrimination at the institutional level as an important risk factor for poor mental health outcomes.

## Public health implications

Despite recent interest, the field of public health has not developed a coordinated strategy to quantify and study law enforcement overexposure and violence as a public health issue. Our findings support preliminary evidence of an association between persistent police exposure and poor mental health status. This is a salient finding given the apparent pressure the Chicago Police Department is under to increase the number of street stops (WBEZ, 2016). In 2014, the rate of stop and frisk in Chicago was four times higher than the rate in New York City during the height of their stop and frisk program (American Civil Liberties Union of Illinois, 2015). Given the current political climate and potential return to law-and-order policing, we need renewed focus and momentum to understand and address the potential health implications of persistent and aggressive policing.

## Availability of data and materials

Sinai Community Health Survey 2.0 datasets, questionnaires and methodology report are available via application to the Inter-university Consortium for Political and Social Research repository.

## Appendix

**Table A1.** Characteristics of the study population compared with Chicago and the USA

	Study population^a^	Surveyed communities^b^	Chicago^b^	USA^b^
Male (%)	50	50	49	49
Median age (years)	40	31	34	38
Non-Hispanic Black (%)	29	29	31	12
Non-Hispanic White (%)	16	15	32	62
Hispanic (%)	54	54	29	17
Mexican (%)	43	43	22	11
Puerto Rican (%)	6	7	4	2
High school graduates (%)^c^	71	69	83	87
Unemployment rate (%)^d^	14	14	11	7

a Sinai Community Health Survey estimates were weighted to account for complex sampling design.

b 2012–2016 American Community Survey.

c Among those 25 years or older.

d Among those 18 years or older in study population compared with 16 years or older in surveyed communities, Chicago and USA.
